# Inferior Alveolar Plus Buccal Nerve Block Decreases Postoperative Pain Scores at Buccal Mucosal Graft Harvest Site: A Retrospective Analysis

**DOI:** 10.5152/tud.2023.23080

**Published:** 2023-09-01

**Authors:** Vivek Tarigopula, Swarnendu Mandal, Gorrepati Rohith, Abhay S. Gaur, Manoj K. Das

**Affiliations:** Department of Urology and Renal Transplant, All India Institute of Medical Sciences, Bhubaneswar, India

**Keywords:** Inferior alveolar nerve, buccal nerve, buccal mucosa, nerve block, urethroplasty

## Abstract

**Objective:**

Postoperative pain at buccal mucosal graft (BMG) harvest site hinders the resumption of food intake. We aim to study the effect of inferior-alveolar nerve block plus buccal nerve block (IANB + BNB) on pain scores.

**Methods:**

This was a retrospective case–control study performed in a single center from July 2021 to July 2022 (ethics committee approval: T/IM-NF/Urology/23/27). We performed IANB + BNB with a mixture of 5 mL each of 1% lignocaine and 0.25% bupivacaine and 4 mg dexamethasone, in addition to local infiltration of 2% lignocaine and (1:100 000) epinephrine combination before harvesting BMG. We retrospectively compared the recorded postoperative pain scores using the visual analog scale (VAS) among patients who received and did not receive IANB + BNB. The time for resumption of pain-free diets and postoperative analgesic requirements was compared.

**Results:**

The study groups included 20 patients each and were similar in age and graft size. The VAS scores at 0 hours [1.0 (1.25) vs. 2.5 (3.5); *P=* .043], 6 hours [2.40 (± 0.69) vs. 4.60 (± 0.97); *P=* .008], 12 hours [2.50 (± 0.97) vs. 4.80 (± 0.92); *P=* .008], and 24 hours [3.0 (1.25) vs. 4.5 (1.25); *P=* .002] were better in the intervention arm. However, the pain beyond the second day was similar. The IANB + BNB group resumed solid food quicker, and the cumulative paracetamol dose required was less [8.9 (± 3.03) vs. 16.2 (± 5.06) g; *P=* .001]. Fewer patients required opioids.

**Conclusion:**

Patients who received IANB + BNB had better pain scores during the first 24 hours following surgery and tolerated solid diet quicker.

**MAIN POINTS**

- The inferior alveolar plus buccal nerve block decreases postoperative pain at the buccal mucosal graft harvest site.
- The effect of better pain scores lasts for at least the first 24 hours following the surgery.
- Patients who received the block had lesser postoperative analgesic requirements and opioid consumption.
- Patients who received the block resumed pain-free solid diets quicker by 1 day.

## Introduction

Buccal mucosal graft (BMG) is the most common and versatile graft used for various techniques of urological reconstruction. The BMG harvest for various kinds of urethroplasties, which is a simple and commonly performed procedure world over, is not without its own complications.^1,2^ Postoperative pain at the BMG harvest site often hinders the resumption of food intake.^3^

Various strategies to alleviate pain, like infiltration of the donor site with various types of local anesthetics and primary closure of the donor wound, have been tried in the past.^3–8^ With regard to primary wound closure, which is the most commonly used and most extensively studied technique, the existing literature shows contradicting outcomes in terms of patients’ postoperative morbidity. Hence, no clear-cut, proven strategy to tackle this issue is available at the current date.

Patients undergoing BMG harvest at our center often report that the pain at the harvest site is more bothersome than at the surgical site. Pain, in turn, hampers oral intake post-harvest. There have been instances where the patient’s postoperative hospital stay has been extended for this same reason. We explored possible ways to help our patients and enhance their postoperative course. 

The combined inferior alveolar and buccal nerve block (IANB + BNB), which is commonly used in lower jaw dental procedures, covers the area of interest of BMG.^9,10^ The inferior alveolar nerve supplies buccal mucosa over the first premolar, canine, and lower incisors. The buccal nerve innervates the buccal mucosa over the second premolar, lower molars, and gingiva. This block can potentially alleviate pain after buccal nerve harvest. Upon trying this procedure on a couple of patients after consent, we found positive results. We started adopting this approach as a common practice at our institute. To our knowledge, the application of the combined IANB + BNB for BMG harvest is yet to be studied. We aim to study the effect of IANB + BNB on postoperative pain scores at the BMG harvest site.

## Material and Methods

### Study Design

This case–control study was performed in the department of urology at a single tertiary care center in India. Informed consent was obtained from all the study participants. An institutional ethical committee approval (All India Institute of Medical Sciences Bhubaneswar) was obtained (Reference number: T/IM-NF/Urology/23/27). The study duration was from July 2021 to July 2022. 

### Study Subjects

**Figure 1.**
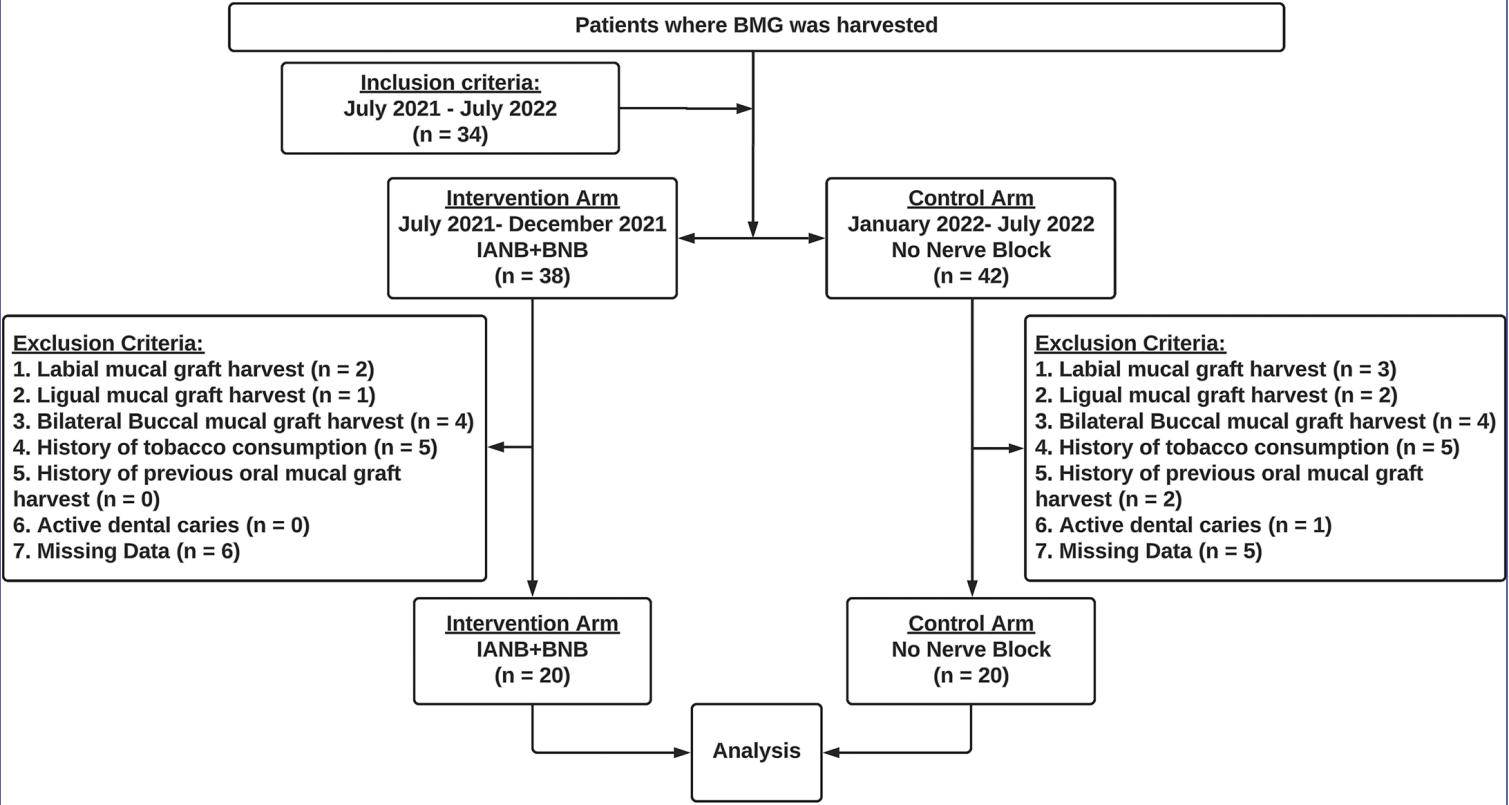
Study flowchart. IANB + BNB, inferior alveolar nerve block plus buccal nerve block.

All the patients who underwent BMG harvesting were considered for the study from July 2021 to July 2022. The patients who underwent BMG harvest between July 2021 and December 2021 underwent the procedure without any nerve blocks. From January 2022 to July 2022, all the patients underwent the procedure after IANB + BNB. Patients with additional labial/lingual mucosal graft harvest, a history of tobacco consumption, previous history of BMG harvest, active dental infections, and poor oral hygiene, and patients with missing data were excluded. The flowchart of the study is shown in Figure 1.

### Study Procedure

All the patients were preoperatively instructed on twice-daily brushing and chlorhexidine mouthwash thrice-daily, along with oral hygiene. All the surgical steps were standardized. All the patients underwent nasal intubation to facilitate BMG harvesting.

### Intervention

After painting and draping, the oral cavity of the patient was opened and held in position using a Doyen’s mouth gag. The desired length of BMG to be harvested was marked on the cheek after identifying and excluding Stenson’s duct opening. 

In the study arm, IANB + BNB was performed. A mixture of 5 mL of 1% lignocaine, 5 mL of 0.25% bupivacaine, and 4 mg of dexamethasone were prepared. A 24-gauge needle with a syringe was taken. 

For IANB, the needle was inserted parallel and about 1 cm above the mandibular occlusion plane toward the pterygomandibular raphe until the ramus was hit. Then, the needle was withdrawn slightly for about a millimeter and aspirated to rule out intravascular placement. Then 4-5 mL of the block were injected. For BNB, the needle is withdrawn and reinserted just lateral and anterior to the edge of the ramus at the level of occlusion of the posteriormost molar. The needle was advanced for 3-5 mm, and about 4 mL of the block were injected. 

Following this, local infiltration with a 1:1 combination of 2% lignocaine and (1:100 000) epinephrine was injected submucosally. This was used to hydro-dissect and lift the mucosa to facilitate harvest. The size of the graft required depended on the type of procedure and the index case. Once the required size of BMG was harvested, the harvest site was left to heal by secondary intention, and no wound closure was performed. The harvest site was packed with gauze soaked with 1:100 000 epinephrine immediately after the harvest for 6-8 hours. The pack removal was performed on postoperative day (POD) 1, along with cold water irrigation. Any bleeders were checked visually. 

The control arm comprised patients who did not receive any nerve block between July 2021 and December 2021. It is because we changed our practice to incorporate performing the IANB + BNB in patients requiring BMG from January 2022. The graft was harvested after injecting a 1:1 combination of 2% lignocaine and (1:100 000) epinephrine. This was injected in a plane beneath the mucosa. The remaining procedure was similar to the study arm. 

Oral rehabilitation of the patients started once the pack was removed, and the patients were encouraged to resume cold liquids initially, followed by a solid diet as tolerated. Intravenous paracetamol was used as the postoperative analgesic, and opioids in the form of tramadol 100 mg were used for breakthrough pain. 

### Parameters Assessed

Initial baseline and demographic factors were recorded using a prespecified case record proforma. Postoperative pain scores at the harvest site were recorded using the visual analog scale (VAS). The values were recorded at intervals of 0 hours, 6 hours, 12 hours, 24 hours, 48 hours, and every 24 hours until POD 5 or discharge, as a *common* practice in our department. The time of resumption of pain-free liquid and solid diet intake was also recorded. 

### Statistical Analysis

The categorical data were expressed using proportions. The continuous data were expressed using mean (standard deviation) or median (interquartile range) based on the normalcy of the data. We analyzed the pain scores, time of resumption of food, and analgesic requirements. A *P*-value of <.05 was considered significant. For comparing parametric data, the independent samples *t*-test was used, and for non-parametric data, the Mann–Whitney test was used. All the statistical functions were performed using the software Statistical Package for Social Sciences version 29.0 (IBM SPSS Corp.; Armonk, NY, USA). 

### Results

A total of 42 patients had BMG harvests between January 2022 and July 2022, and all of them underwent the BMG harvest using IANB + BNB. Thirty-eight patients underwent BMG harvest between July 2021 and December 2021 without any block. Among both groups, after exclusion (the reason mentioned in Figure 1), 40 patients were included in the final analysis (20 each in the intervention arm and control arm). The lack of complete data was the most common reason for the exclusion in both groups. (Figure 1) . 

About 80% of patients (n = 16) in the study arm and 70% (n = 14) of patients in the control arm were male. In the study arm, 40% of patients underwent the procedure for male urethral stricture disease and hypospadias repair, while 20% of patients were females with urethral stricture. In the control arm, 60% (n = 12) were males with urethral stricture disease, 30% (n = 6) were females with urethral stricture, and 2 patients had a hypospadias repair. Both study groups did not differ in terms of age [30.20 (±13.52) vs. 42.8 (±14.15); *P=* .057]. The median BMG size harvested was also not statistically different amongst the two groups [3.75 (2.88) vs. 2.5 (3.5); *P=* .85] (Table 1).

**Figure 2.**
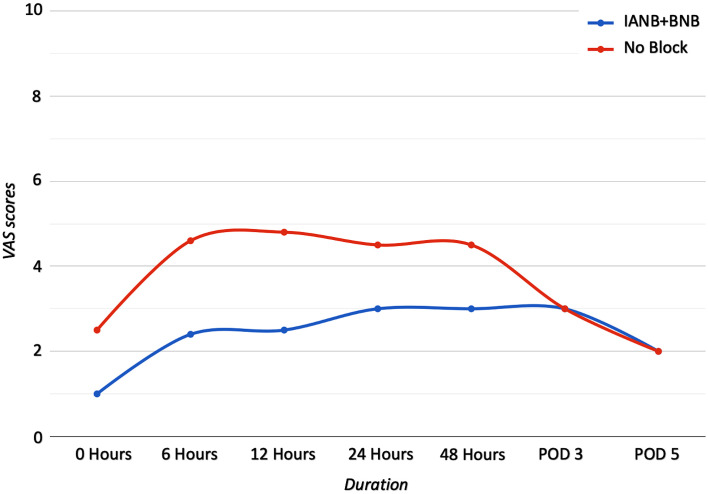
Line graph showing VAS scores in postoperative period. IANB + BNB, inferior alveolar nerve block plus buccal nerve block; POD, postoperative day; VAS, visual analog scale.

On comparing the postoperative pain scores (Table 2, Figure 2), it was observed that VAS scores at 0 hours [1.0 (1.25) vs. 2.5 (3.5); *P=* .04], 6 hours [2.40 (± 0.69) vs. 4.60 (± 0.97); *P=* .008], 12 hours [2.50 (± 0.97) vs. 4.80 (± 0.92); *P=* .008], and 24 hours [3.0 (1.25) vs. 4.5 (1.25); *P=* .002] were significantly less in patients who received IANB + BNB than those who did not receive a block. However, VAS scores at 48 hours on POD 3 and POD 5 were not significantly different. 

Both the study groups were able to resume normal liquid diet by the second POD, while the patients receiving IANB + BNB were able to resume normal solid diet quicker [2.5 (1.25) vs. 3.5 (1.25) days; *P=* .009]. The paracetamol dose consumed during the first 5 days of surgery was significantly less in patients who received IANB + [8.9 (±3.03) vs. 16.2 (±5.06) g; *P=* .001]. About 30% of patients required at least 1 dose of opioid for breakthrough analgesia as opposed to 50% of patients who did not receive a block.

## Discussion

Donor site morbidities following BMG harvest have been studied previously.^2^ Important morbidities of this procedure are postoperative pain at the harvest site, scarring, contracture, and difficulty in mouth opening.^11^ There is a rare chance of hematoma formation if the wrong plane is entered and hemostasis is not done properly.^12^ Perioral numbness is an additional complication if the mental nerve is injured while harvesting labial mucosa.^13,14^ In contrast, perioral numbness following BMG harvest was also reported in 68% of patients by Wood et al.^3^ The authors have commented that this was a consequence of the excision of the mucosa and is unavoidable. Persistent changes in salivary secretion have been reported in 11% of patients in the same study. 

Significant postoperative pain often hinders the resumption of a normal oral diet. As many as 83% of the patients considered BMG harvest painful, and more than half of them thought that the pain at the harvest site was worse than expected.^3^ In a study by Dublin and Stewart,^1^ out of 35 patients who have undergone BMG harvest, more than 64% of patients reported being in pain at the donor site even 48 hours following the graft harvest. Various strategies to alleviate pain have been tried with variable success. 

In the same retrospective analysis, primary wound closure after graft harvest was found to be more painful, especially until POD 5.^3^ In a randomized controlled trial (RCT) by Muruganandam et al,^4^ a total of 50 patients were randomized 1:1 to either undergo wound closure or non-closure. They found that donor site pain was significantly less in patients with non-closure on POD 1 and 2. In a similar RCT by Rourke et al^5^ in 2012, a total of 50 patients were randomized to either wound closure or non-closure. They found that the non-closure group had better pain scores till POD 3, following which the pain slightly worsened compared to the closure group. The non-closure group also had an earlier return to a regular solid diet (70.8% vs. 19.2% on POD 1) and full mouth opening (79.1% vs. 15.3%). The authors postulated that wound closure under tension causes more pain owing to edema incited by wound closure. Barbagli et al^15^ have postulated that an ovoid shape of the graft may aid in tension-free wound closure as opposed to a rectangular-shaped graft. They further commented that increased postoperative pain might be more so because of the rectangular shape of the graft rather than closure or non-closure. Similar results were seen in another RCT by Soave et al^6^ in 2018, where 135 patients were included. They concluded that wound non-closure was non-inferior to the primary closure of the donor site in terms of postoperative pain and morbidity. Contrary to the earlier findings, an RCT by Wong et al,^7^ comprising 34 patients, found that the pain scores were comparable, but the closure group could tolerate a regular diet earlier. 

Chua et al^8^ in 2020 published an RCT investigating a new strategy. They randomized 50 patients to either receive 2% lignocaine with epinephrine as hydro dissection (control arm) or an additional injection of 20 mL 1.3% liposomal bupivacaine injection at the muscle bed and mucosal edges (intervention arm). They found out that postoperative pain scores at the donor site were comparable. Still, narcotic use in the first 24 hours of surgery decreased in the intervention arm, though the difference did not persist at 48 hours. 

Jonnavithula et al^16^ have tried the applicability of nerve block in urethroplasties for the first time. They found infraorbital nerve block superior to no block in terms of postoperative pain scores and early resumption of diet. In this RCT comprising 30 patients, pain scores were better in patients who received block, though the exact duration of how long the effect lasted was not mentioned. Moreover, we feel that the denervation of the infraorbital nerve does not completely cover the area of interest of a BMG. Its effect has previously been studied in cleft lip repair, nasal, and transsphenoidal surgeries.^17–19^

The combined IANB + BNB, commonly used in lower jaw dental procedures, covers the area of interest of BMG but has not been studied in this setting to our knowledge. The inferior alveolar nerve, which arises from the mandibular nerve, supplies sensory innervation to the ipsilateral half of the mandible, the buccal mucosa over the first premolar, canine, and lower incisors, and the skin over the chin and ipsilateral lower lip.^9^ The buccal nerve innervates the buccal mucosa over the second premolar and lower molars and the gingiva.^10^ The procedure of applying the IANB + BNB is relatively straightforward once the surgeon becomes familiar with the regional anatomy.^20^

In our study, we found that patients receiving IANB + BNB had better pain scores during the first 24 hours of the block and could resume solid diet intake quicker by about 1 day. It was also seen that postoperative analgesic use and the requirement for opioid analgesics have decreased. However, some patients required prolonged analgesics to control pain at the operation site rather than the pain at the donor site.

This study is the first of its kind where the usage of IANB + BNB has been studied in the setting of reconstructive urology, especially where BMG harvest is required. To our knowledge, no such study has been done in the existing literature.

### Limitations

This study, though encouraging, has its limitations. There was no formal sample size calculation done. This was a retrospective study, and all the required data was unavailable for all the patients, resulting in the exclusion of a few patients. A prospective randomized study with a larger sample size would have better eliminated the biases regarding the type of surgery for which the BMG was harvested and other unknown confounding factors. Other morbidities at the donor site were not assessed completely due to the lack of complete data availability. 

Patients who received IANB + BNB had better pain scores at the BMG harvest site when compared to patients who did not receive a nerve block during the first 24 hours following surgery. This enabled them to resume a normal solid diet more quicker. This effect did not last after the first POD. We can conclude that by applying this easily reproducible strategy, patients undergoing urethral reconstruction can benefit the world over.

## Figures and Tables

**Table 1.** Baseline Patient Characteristics

**Parameter**	**IANB + BNB**	**No Block**	***P***
Number of patients	20	20	
Gender			
Male	16	14	
Female	4	6	
Indication for surgery			
Male urethral stricture	8	12	
Female urethral stricture	4	6	
Hypospadias	8	2	
Mean age (±SD) in years	30.20 (±13.52)	42.8 (±14.15)	.057
Median graft size (IQR) in cm	3.75 (2.88)	2.5 (3.5)	.85

IANB + BNB, inferior alveolar nerve block plus buccal nerve block, IQR, interquartile range; SD, standard deviation.

**Table 2.** Postoperative Parameters

**Parameter**	**IANB + BNB**	**No Block**	***P***
VAS at 0 hours [median (IQR)]	1.0 (1.25)	2.5 (3.5)	**.04**
VAS at 6 hours [mean (±SD)]	2.40 (±0.69)	4.60 (±0.97)	**.008**
VAS at 12 hours [mean (±SD)]	2.50 (±0.97)	4.80 (±0.92)	**.008**
VAS at 24 hours [median (IQR)]	3.0 (1.25)	4.5 (1.25)	**.002**
VAS at 48 hours [median (IQR)]	3.0 (1.25)	4.5 (2.0)	.19
VAS on POD 3 [median (IQR)]	3.0 (2.0)	3.0 (1.25)	.08
VAS on POD 5 [Median (IQR)]	2.0 (1.25)	2.0 (1.00)	.10
Median duration (IQR) of normal liquid diet days	1.5 (1.0)	2.0 (0)	.14
Median duration (IQR) of normal solid diet days	2.5 (1.25)	3.5 (1.25)	**.009**
Mean (±SD) paracetamol requirement in grams	8.9 (±3.03)	16.2 (±5.06)	**.001**

IANB + BNB, inferior alveolar nerve block plus buccal nerve block; IQR, interquartile range; POD, postoperative day; SD, standard deviation; VAS, visual analog scale; bold values, statistically significant.
